# Emergence of early alterations in network oscillations and functional connectivity in a tau seeding mouse model of Alzheimer’s disease pathology

**DOI:** 10.1038/s41598-017-13839-6

**Published:** 2017-10-27

**Authors:** A. Ahnaou, D. Moechars, L. Raeymaekers, R. Biermans, N. V. Manyakov, A. Bottelbergs, C. Wintmolders, K. Van Kolen, T. Van De Casteele, J. A. Kemp, W. H. Drinkenburg

**Affiliations:** Department of Neuroscience Discovery, Janssen Research & Development, a Division of Janssen Pharmaceutica NV. Turnhoutseweg 30, B-2340 Beerse, Belgium

## Abstract

Synaptic dysfunction and disconnectivity are core deficits in Alzheimer’s disease (AD), preceding clear changes in histopathology and cognitive functioning. Here, the early and late effects of tau pathology induction on functional network connectivity were investigated in P301L mice. Multichannel EEG oscillations were used to compute (1) coherent activity between the prefrontal cortex (PFC) and hippocampus (HPC) CA1-CA3 networks; (2) phase-amplitude cross frequency coupling (PAC) between theta and gamma oscillations, which is instrumental in adequate cognitive functioning; (3) information processing as assessed by auditory evoked potentials and oscillations in the passive oddball mismatch negativity-like (MMN) paradigm. At the end, the density of tau aggregation and GABA parvalbumin (PV+) interneurons were quantified by immunohistochemistry. Early weakening of EEG theta oscillations and coherent activity were revealed between the PFC and HPC CA1 and drastic impairments in theta–gamma oscillations PAC from week 2 onwards, while PV+ interneurons count was not altered. Moreover, the tau pathology disrupted the MMN complex amplitude and evoked gamma oscillations to standard and deviant stimuli suggesting altered memory formation and recall. The induction of intracellular tau aggregation by tau seed injection results in early altered connectivity and strong theta–gamma oscillations uncoupling, which may be exploited as an early electrophysiological signature of dysfunctional neuronal networks.

## Introduction

Alzheimer’s disease (AD) is a progressive, neurodegenerative disease characterized by cognitive and behavioral alterations including deficits in attention, learning and memory, information processing, circadian rhythms and sleep maintenance^1,2^. A key feature of AD is a dual proteinopathy including extracellular senile plaques consisting of amyloid-β (Aβ) peptides^3^ and intracellular neurofibrillary tangles (NFTs), composed of abnormally hyperphosphorylated aggregated tau protein^4^. The pathology and neuronal loss are cardinal features of AD, which invariably affect multiple brain regions. Brain atrophy emerges in asymptomatic individuals, with the first identifiable changes occurring nearly a decade before severe cognitive symptoms and dementia^5^. While the exact underlying mechanisms that give rise to impairments in diverse behavioral and cognitive domains are unclear, in general, the regional presence of NFTs parallels the regional neurodegeneration and associated clinical symptoms presented by the patients^6,7^.

Evidence suggests that synapses and cell losses that ensued abnormal neural oscillations and network disconnectivity may be a common denominator that contributes to the cognitive and behavioral alterations associated with AD^8–10^. Neuronal oscillations facilitate synaptic plasticity and play a key role in transient, long-range communication of distinct brain regions^11^. In the hippocampus, two main oscillations reflect physiological synchronous activities, both being implicated in behavioral states and memory performance: theta oscillations (4–12 Hz), generated by synchronous phasic firing of pyramidal cells, and gamma oscillations (25–100 Hz), generated by circuits between GABAergic interneurons and pyramidal cells^12^.

Reduced amplitudes of EEG oscillations in the alpha and gamma frequency range, and enhanced amplitudes of delta and theta oscillations represent the earliest alterations of local network oscillations described in AD patients^13,14^. Alterations are also detectable as failure of functional interactions between multiple brain areas, which was revealed by pathological reduction in coherent alpha activity between brain regions of AD patients^15–17^. Amyloid plaque deposition is most prominent in the default-mode network (DMN), a network of brain areas where metabolism is preferentially elevated^18,19^. Interestingly, disturbed large-scale network activity has been confirmed in neuroimaging studies, especially with respect to a reduced deactivation of the DMN during resting periods following cognitive tasks^20–22^. AD patients with the sporadic form of the disease are particularly susceptible to seizures with a varying prevalence rate ranging from 10 to 22%^23,24^. Frequent epiletiform discharges are more frequent in patients with early disease onset^25,26^ and increases with progression of the disease^27^.

The progressive loss of synapses and network abnormalities that occur in AD is also observed in animal models of dementia. Several mouse models that have been developed are good models for certain aspects of AD disease but do not completely mimic the human AD brain pathology. These models have provided some insight into the neurobiology of the protein products of AD-related genes, the ways in which mutations cause dysfunction and the effects of pathology on other pathways, which is useful for proof of concept pharmacological testing of disease interception therapy^28^. Disruption in hippocampal oscillations has been correlated with an increase of Aβ level and the appearance of plaques^29^, however changes in hippocampal and cortical network oscillations may occur earlier prior Aβ plaque loads^9,10,30^. Accordingly, various factors have been hypothesized to influence the phenotype such as the timing of testing and treating animals as the age at which each model develops certain aspects of the AD pathology varies. Interestingly, the various transgenic mice exhibit disruption in theta rhythmicity; of which a number of them present cognitive decline associated with an increase in theta activity^31,32^, whereas others displayed reduced theta rhythm^29,33,34^. Although recent studies have provided novel insights into the pathogenesis of AD, detailed information is still lacking on how the dysfunctional septohippocampal connectome results in enhanced or impaired theta rhythmicity and neuronal hyperexcitability in AD. In addition, alterations in network activities in AD mouse models of amyloidosis are accompanied by an early imbalance in inhibition and excitation, which overall leads to hyperexcitability-induced seizures^9,35^.

Early indicators of AD are crucial for implementing interventions while the brain systems are still relatively normally functioning. Here, the transgenic P301L mouse^36^ has been used to examine the effects of pre-formed fibril (PFF) tau seeding on the activity of neuronal circuits before tau pathology. Afterwards, it was investigated whether subtle memory information processing deficits may be detectable before neuronal loss or tangle deposition occurs. We have used the K18 seed fragment infused into P301L mouse seed model^37^ to explore whether fine alterations in intra-hippocampal (HPC) and prefrontal cortex (PFC) network oscillations and connectivity might be detectable at very early stages to potentially provide some explanation for electrophysiological alterations that may be associated with the progression of tau pathology and neuronal loss observed in mouse model of taupathies. In the first study, longitudinal 24-hrs recordings of the sleep-wake cycle and related spectra were performed in mice chronically equipped with cortical epidural EEG/EMG electrodes. In the second study, longitudinal functional network recordings were carried out in mice equipped with multiple EEG electrodes to investigate 1)-coherent activities between PFC and HPC CA1-CA3 networks; 2)-the level of phase-amplitude cross-frequency coupling between theta and gamma frequency oscillations within those brain networks; 3)-information processing during auditory evoked potentials and evoked oscillations. Additionally, the accumulation of aggregated phospho-Tau and the density of GABAergic PV+ interneurons were quantified by immunohistochemistry.

## Results

### Vigilance states EEG Spectra

**Figure 1:**
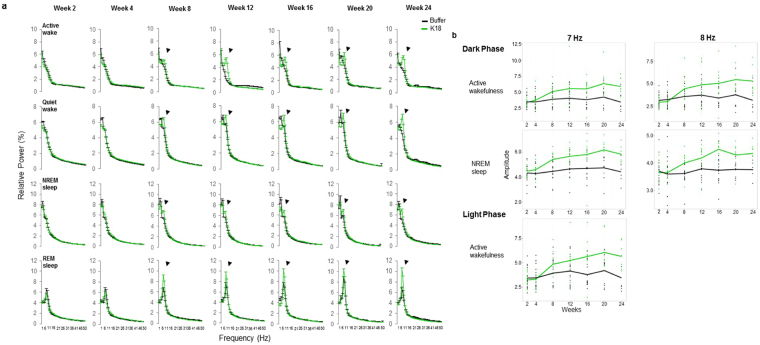
(**a**) Cortical (epidural) mean relative EEG power spectra during waking, NREM sleep and REM sleep in buffer controls (black, n = 11, 4 animals were excluded due to mislocation of the injection and electrode sites or signal artefacts) and K18 (green, n = 15) conditions. Note the characteristic increases in relative theta amplitude after the administration of K18, which is clearly evident in all vigilance states starting from 8 weeks post-treatment onwards. Data are presented as mean values ± SEM for buffer (black) and K18 (green) conditions. **(b)** longitudinal changes in the amplitude at frequency bins centered at 7 and 8 Hz with the time during active waking and NREM sleep as observed in K18 treated animals. ANOVA with post hoc Hochberger’s and Dunnett’s tests.

In the first study, the most consistent overall effects of K18 aggregate were observed in the amplitude of cortical EEG spectra. Prominent increases in relative theta amplitude were found in all vigilance states (p < 0.05) (Fig. 1a and Suppl Fig. 1a), starting at 8 weeks post-HPC CA1 infusion of K18, which coincides with the first signs of neuronal death^37^ after which it remained stably higher up until 24 weeks.

Decreases in the relative power below 4 Hz were observed during quiet waking from 2 to 20 weeks. Subsequent  longitudinal analysis of the changes in amplitudes in the first 20 frequency bins as a function of time showed a consistent increase at frequency bins centred at 7 and 8 Hz during the dark phase for active waking (for slope p = 0.001 and p = 0.004, respectively), and for NREM selle (for slope p = 0.028, respectively).  Similar increases in peak frequency at 7 Hz were observed during the light phase of the circadian time (p = 0.004) (Fig. 1b). Therefore, the end-point of the second functional network study was set at 20 weeks.

### Multichannel Network Oscillations

#### Effect of K18 on EEG oscillations

**Figure 2:**
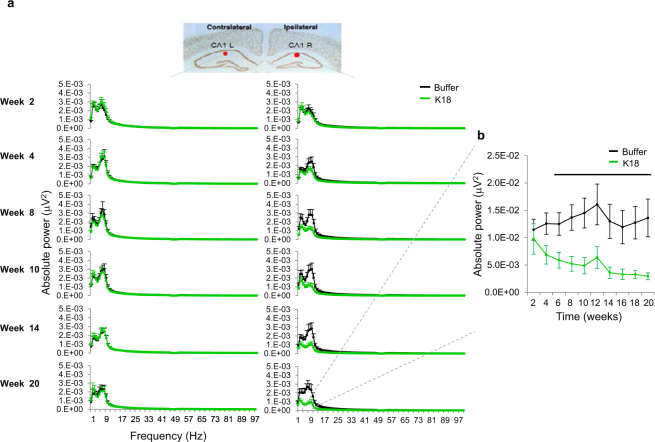
(**a**) Longitudinal mean power spectra in 1–100 Hz from HPC-CA1 injection ipsilateral and their contralateral sites over the second week through to the last recordings (weeks 20) post-administration of buffer (black) and K18 (green). (**b)** Early drop of the power spectra in the slow theta frequency band starting on week 4, which became strong on week 20. Data are presented as mean values ± SEM and black bar above curves indicates interval of significant level difference between buffer (black, n = 9) and K18 (green, n = 8) groups. 3 animals across different conditions were discarded from the analysis because of signal artifacts.

Spectral characteristics during the dark phase of the HPC CA1 in the buffer condition and following K18 infusion are presented in Fig. 2a. K18 consistently reduced the power of the theta frequency range in the HPC CA1 in the injection side from 4 weeks post-injection (F(1,14) = 4.5, p = 0.05) and remained consistently lower from week 8 (F(1,14) = 6.1, p = 0.02) till the end of the experiment at week 20 (F(1,14) = 6.08, p = 0.02). In addition, the evolving tau aggregation was associated with persistently lowered hippocampal slow delta band amplitude from 8 weeks onwards. However, EEG spectra were unaltered in higher frequency oscillations.

#### Effects of K18 on networks coherence

**Figure 3:**
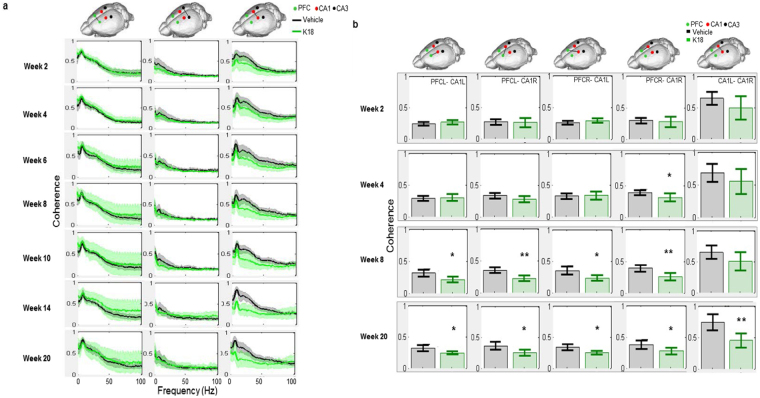
(**a**) Coherence patterns in contralateral recording sites over 1–100 Hz in buffer controls (black) and K18 (green) conditions from week 2 through to week 20 post-administration. Bar charts showing the mean coherent activity ( ± 95 CI) in the theta frequency range. (**b)** mean coherent activity in the theta frequency range over weeks 2, 4, 8 and 20. Data are presented as mean values ± SEM for buffer (black, n = 9) and K18 (green, n = 8) groups. 3 animals across different conditions were discarded from the analysis because of signal artifacts. Asteriks indicate significant (*p < 0.05, **p < 0.01) differences.

Here, we addressed whether tau aggregation affects network connectivity by measuring pair-wise coherent activity in PFC-HPC CA1-CA3 networks during waking state (Fig. 3a). It was found that coherence in different PFC-HPC pairs is initially dominated by theta, and significant changes occurred in the patterns of intra- and interhemispheric PFC-HPC CA1 coherence as brains progress in pathology (Fig. 3a). To study in detail the dynamics of theta coherence, we averaged coherence within the theta band, and compared paired coherencies over consecutive recordings weeks. We found a decrease in the theta frequency coherent activity in PFC-HPC CA1 in the right K18 injection hemisphere on week 4 (Fig. 3b), followed by a sustained drop in intra- and interhemispheric theta frequency coherence from week 8 onwards (Fig. 3b).

#### Effects of K18 injection on phase-amplitude cross-frequency coupling (PAC)

**Figure 4:**
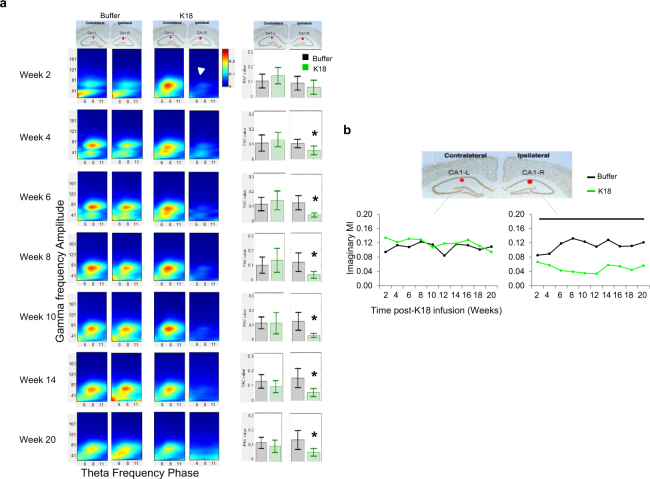
Representative comodulation Phase-Amplitude maps showing early deficit in theta-gamma phase-amplitude coupling during waking state in recordings from the hippocampal CA1 of K18-treated mice. **(a)** Bar panels represent mean modulation index (MI) values ± SEM in mice injected with K18 (green) and buffer (black) showing a significant decreased in coupling strength for K18 infused animals as revealed in theta-gamma MI (p < 0.05). (**b)** Time course of mean MI showing some sustained impairments in the strength of theta-gamma phase-amplitude coupling from the right HPC CA1 of K18 group (green, n = 8) as compared to buffer (black, n = 9) groups. 3 animals across different conditions were discarded from the analysis because of signal artifacts.

Analyses described above demonstrate K18 induced decreases in the EEG theta frequency power relative to baseline and reduced coherent theta-regulated coordination in fronto-hippocampal recoding pairs. To understand the degree of interactions between low and high frequency oscillations, we have computed the phase–to-amplitude coupling (PAC) coupling using the modulation index (MI). Under control buffer conditions, mean MI maps showed that the phase of slow theta frequency oscillations strongly coupled and modulated the amplitudes of high gamma frequency band at HPC CA1 sites in both hemispheres (Fig. 4a left side). Under K18 condition, most of the MI maps (Fig. 4a right side) have clear peaks that occur at a theta frequency in the left HPC CA1 site supporting prominent theta coordination. However, the coupling of theta and gamma were barely visible compared to buffer treatment (p < 0.05) in the HPC CA1 injection site from the first 2 weeks which persisted throughout the 20 weeks follow-up period (Fig. 4a,b), this precedes maximum tau pathology and neuronal loss shown earlier to occur at 4 and 8 weeks, respectively.

### Cognitive Auditory Information Processing: MMN

**Figure 5:**
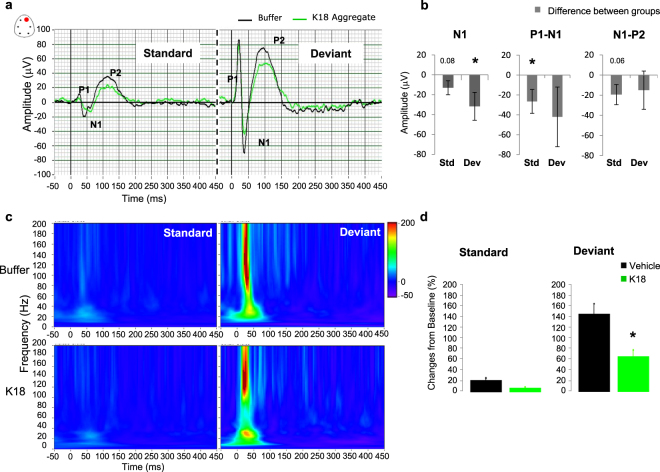
(**a**) Grand averaged auditory evoked waveforms recorded from the frontal right electrodes in the passive oddball (MMN) paradigm with frequency deviants, (**b)** Post-stimuli peaks changes were estimated over all recording sites N1-P1 and N1-P2 complex amplitudes as well as for N1 component’s amplitude, (**c)** the power of evoked oscillations in response to standard and deviant stimuli and (**d)** changes in the evoked 40–80 Hz gamma oscillations. Note the decreased amplitudes and evoked oscillations to both standard and deviant acoustic stimuli may suggest impairments in the encoding and discrimination facilitation of the sound features during information processing. Data are presented as mean values ± SEM for buffer (black, n = 9) and K18 (green, n = 8) groups, 3 animals across different conditions were discarded from the analysis. Mixed effect model ANOVA.

The MMN is an electrophysiological indicator of the pre-attentive stages of short-term memory involved in sensory auditory information processing. In the auditory cortex, a detectable change (deviant stimulus) in the pitch frequency of a repeated (standard) stimulus evoked a prominent negative polarity in the auditory potential component within a 30–50 ms time window following stimulus onset, likely reflecting a MMN-like response (Fig. 5a).

Tau pathology disrupted P1/N1/P2 complex amplitude and evoked oscillations to standard and deviant stimuli (Fig. 5a,b,c,d). Linear mixed model estimates over all the recording sites showed a difference between treatment groups for the N1 peak amplitude of the standard (13.05; p = 0.08) and deviant waveforms (31.7; p = 0.02) (Fig. 5b, left panel). The peak amplitude differences in N1-P1 complex was significantly reduced (−26.5; p = 0.04) for the standard stimuli (Fig. 5b middle panel). The peak-to-peak difference in N1-P2 complex amplitude for the deviant stimuli was also reduced, however not to a level of significance (−19.35; p = 0.06) (Fig. 5b right panel). Thus, K18-injected mice showed lower MMN response than the buffer-treated control mice, which indicates that tau seed material impaired auditory cognitive processing.

The evoked oscillatory activities in the 40–80 Hz were consistently reduced in K18-treated mice during MMN response, particularly to deviant stimuli (F(1,12) = 7.04, p = 0.02) (Fig. 5c and d).

### Tau pathology and GABAergic interneurons PV+ cells

**Figure 6:**
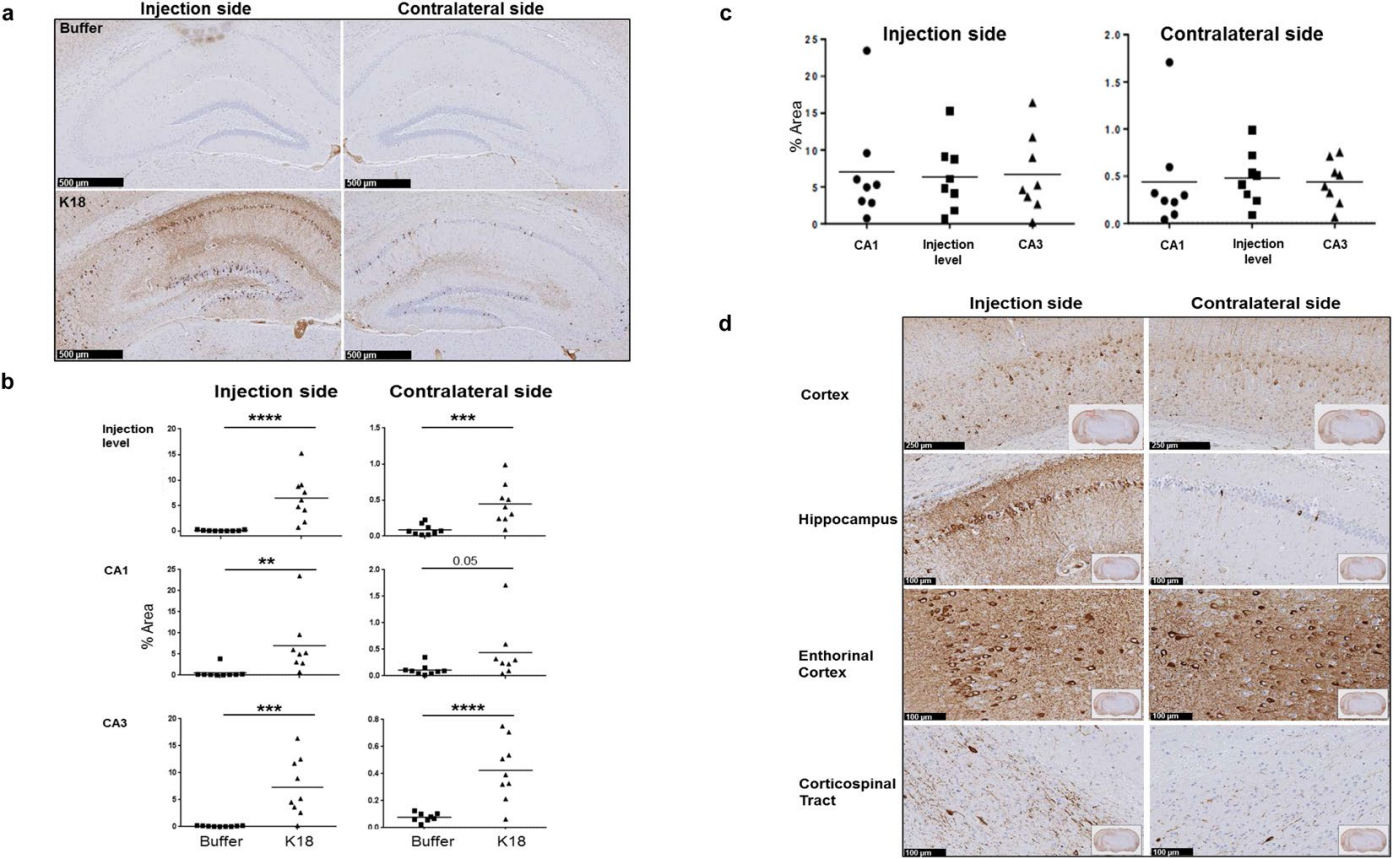
(**a**) AT8 immunoreactivity ipsilateral and contralateral at the level of HPC-CA1 and CA3 structures, scale bars: 500 µm (**b)** Quantification of AT8 labeling revealed strong increases of the tau pathology in different areas of the injection side as compared to the contralateral side, (**c**) total AT8 labeling in the hippocampus and (**d**) distribution of AT8 labeling in the cortex, hippocampus, enthorinal cortex and corticospinal tract. One way ANOVA, Bonferroni correction for multiple comparisons, significance levels p < 0.05, K18 (n = 9) vs buffer (n = 9). 2 animals across different conditions were discarded from the anlaysis because it was difficult to accurately quantify AT8 in some area. Scale bars: cortex: 250 µm; hippocamps, enthorinal cortex and corticospinal tract: 100 µm.

Spatial pattern of the developing tau pathology shows a maximal AT8 labeling in the HPC, ipsilateral to the injection site of K18 (p < 0.0001; CA1, p = 0.007; CA3, p = 0.0001 (Fig. 6a–c). An overall pathology spread was observed throughout the brain; with pathology spreading ipsilateral and contralateral to areas connected to the K18-injection HPC site (Fig. 6b and c). Elevated AT8 immunoreactivity was found in the prefrontal cortex, enthorinal cortex, corticospinal tract and HPC (Fig. 6d).

**Figure 7:**
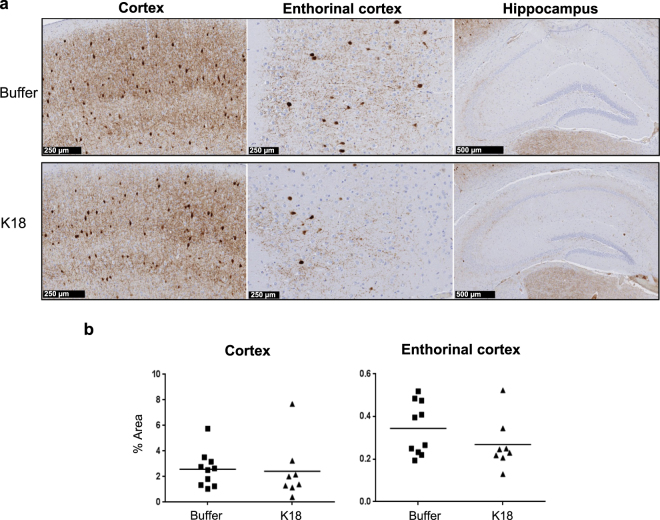
(**a**) Immunohistochemical difference in PV+ cells in hippocampal, frontal and entorhinal cortical structures of mice injected with the buffer (upper panel images) or K18 (bottom panel images). (**b**) Quantification of PV+ cell loss in the cortex and enthorinal cortex. Scatter plot shows no major difference in the average number of PV+ cell loss in cortical structures, whilst limited PV cells were detected in the hippocampus. Accurate quantification of PV+ was difficult in 2 animals, which were discarded from the anlaysis. One way ANOVA, Bonferroni correction for multiple comparisons, significance levels p < 0.05, K18 (n = 8) vs buffer (n = 10). Scale bars 250 and 500 µm.

 PV+ area/total area was calculated in the cortex and enthorinal cortex. As PV+ cells were not detectable in hippocampus, this area was excluded from image analysis. Representative images are depicted in (Fig. 7a). No significant cell loss was found in the cortex and enthorinal cortex (Fig. 7b), whereas limited PV cells were detected in the hippocampus. After finding that GABAergic interneurons PV+ cells were not reduced, whilst pathological tau staining was abundantly present in different brain regions, we have not examined if the pathological tau markers were localized in GABAergic interneurons.

## Discussion

The present study aimed to temporally dissociate the alteration in the activity of neuronal circuit and memory loss with that associated with the pathology induced by exogenous PPF aggregate directly infused in the right HPC CA1 region of P301L mice. We demonstrate the emergence of early network abnormalities expressed by impaired PFC-HPC coherence and HPC theta-gamma PAC, both of which are hallmark rhythms of local and widespread computations as well as altered sleep-wake state-related EEG power theta spectra, associated with late sleep fragmentation.

### Vigilance states EEG spectra and seizures

Disruption in neuronal excitability-inhibitory balance is a potential mechanistic point of convergence for Aβ and tau toxic proteins^38,39^. While neuronal activity declines in the later stages of AD, evidence indicates that it is increased in the early stages of the disease. Functional imaging showed a hyperactive HPC in the mild cognitive impairment stage of AD^40^, and in the asymptomatic preclinical stage of AD^41^. EEG neuronal oscillations play a critical role in memory processes in the hippocampus and are thus hypothesized to also be altered in AD. The hallmark of EEG abnormalities in AD patients is a shift of the power spectrum to lower frequencies, a slowing of theta oscillations and a decrease in coherence of fast rhythms^42,43^. These abnormalities are believed to be associated with functional disconnectivity between cortical areas because of the death of cortical neurons, axonal pathology, and cholinergic deficits^14^. Accordingly, increasing evidence suggests that oscillatory activity in the theta and gamma ranges is altered in AD patients, and changes in theta oscillations are viewed as a possible predictor for AD^13,43^. EEG theta rhythm can be recorded from many areas of the brain, from the lower brainstem to the neocortex, and has been regarded as a “correlate” of arousal, orientation, exploration, attention, learning, memory, voluntary movements, and REM sleep^12,44^. Theta rhythm is strongly dependent on cholinergic neurons in Meynert’s nucleus and also of the ventrolateral nucleus of the thalamus which is controlled by the pedunculo pontine tegmentum nuclei^12,45^. Here, consistent decreases in the HPC EEG theta power emerged at 4 weeks post-K18 injection and persisted throughout the study period. These spectral changes occurred within a time frame, during which complete pathology and irreversible neuronal loss occurs at 4 and 8 weeks after synthetic tau injection, respectively^37^.

An increased risk of epileptiform discharges and spontaneous seizures is more frequent in early AD stages, likely attributed to early failure in active inhibitory mechanisms, which results in disinhibition that destabilizes network oscillatory activity^9,25,46,47^. Abnormal function of corticothalamic network has been described in many mouse models of AD that have amyloidosis^9^. However, tauP301L mice also showed network instability and electrophysiological hyperexcitability prior to tangle deposition or neuronal death, likely related to disruption in GABAergic transmission^48^. Abundant astrogliosis and microgliosis markers of an early inflammatory response were observed after the injection of K18 suggesting a role for inflammation in the pathogenic mechanism^37^. Reactive astrocytes are commonly found in putative epileptic foci and the common final pathway linking systemic inflammation to seizures appears to be a loss of cerebrovascular control of brain homeostasis and alteration of the blood-brain barrier (BBB) permeability^49,50^. Here, our EEG and behaviour observations did not reveal generalized epileptic activity progressing into full tonic-clonic seizures, suggesting a functional BBB in K18-treated mice; however, the high level transitions from sleep to waking, likely related to dysregulation of corticothalamic activity, may have occurred, and consequently impaired sleep maintenance (Suppl Fig. 1b). The fact that EEG power spectra are altered early in the K18-injected site where spatial information encoding is processed, implies that connectivity measures relevant to that mechanism are altered. We therefore predicted that PFC-HPC coherence and PAC coordination patterns would change over the course of the tau pathology.

### Theta-gamma phase-amplitude cross-frequency coupling (PAC)

In the HPC, theta and gamma frequency oscillations reflect physiological synchronous activities implicated in behavioral states and are vital for memory and learning performance. Both rhythms are generated by synchronous phasic firing of pyramidal and by circuits between GABAergic interneurons and pyramidal cells, respectively^12^. Further levels of interconnectivity have been revealed through a phenomenon called theta-gamma oscillations PAC, where slow theta frequency oscillations modulate the activity of gamma frequency oscillations. PAC has been proposed as a plasticity mechanism for communication within and between anatomically dispersed neuronal cell assemblies by coordinating the timing of neuronal firing activity in large-scale brain networks required for effective motor and cognitive processing such as memory, learning and attention^51,52^.

The HPC CA1 is crucial for spatial memory formation and this region integrates synapses associated with memory and expectations as well as the memory to be encoded. The HPC CA1 receives distinct afferents from CA3 and entorhinal cortex. These inputs carry different kinds of spatial information that CA1 pyramidal cells must integrate and segregate. EC-CA1 synapses are associated with the current information that is to be encoded, whereas CA3-CA1 synapses are more associated with memory and expectations. Theta-gamma PAC in the CA1 is a key mechanism coordinating integration and segregation of neural activity and information when a subject is challenged to discriminate and to selectively use memorized and current information.

In the rTg4510 mouse model of tauopathy, deficits in the membrane potential oscillations and firing rate of neocortical pyramidal cells occurs prior to significant cell death^53^. In the Aβ mouse model of AD, impairments of theta-gamma PAC arise before Aβ accumulation^54^, which suggests that altered plastic mechanisms of PAC may represent an early event of the pathogenesis eliciting behavioral and cognitive impairments in both AD models. Interestingly, the strength of theta-gamma frequency PAC depends on the theta power and the stronger coupling has been observed in contralateral HPC sites with greater mean theta amplitude in the buffer group. However, the HPC CA1 theta-gamma frequency PAC was strongly deficient in the injection side as from week 2 post-injection of tau PPF seed (corresponding to 50% maximal level of tau aggregation^37^ onwards, which suggests that tau aggregation impaired the strength of theta-gamma oscillations PAC leading to synaptic plasticity failure in the HPC^55^. The decreases in theta power may disrupt the ability of neural networks to dynamically adjust the amplitude of high frequency oscillations, whereas the increased mean theta-gamma oscillations PAC observed in the HPC CA1 left side of K18 treated mice can be explained as the compensatory features described in the above telemetric study. Overall, the present data suggest a causal relationship between the experimentally induced tau aggregation and dysfunctional hippocampal theta-gamma coupling.

### Coherence

Coherence is introduced to measure the pair-wise normalized synchrony and the strength of the functional interconnections between two EEG signals in different frequency domains^55,56^. Coordinated oscillations are fundamental for behavior and cognition, as they govern communication between widespread neuronal networks processing distinct information. Prefrontal cortex, hippocampal CA1 and CA3 are cardinal brain regions involved in working memory, decision making and spatial memory information processing^57^. The neuronal projection from the HPC, principally originating from the ventral HPC including the ventral CA1 and the ventral subiculum regions, directly targets the PFC, and is referred to as the HPC-PFC circuit^58^. Effective communication in this circuit is believed to depend on theta and gamma oscillations; during working memory performance, neuronal firing and coordinated network activity, which are observed in the HPC and PFC structures of many species, reflecting an involvement of dynamic interaction in the HPC-PFC circuit^12,44,58^. The presence of a significant temporal impairment in the pattern of hippocampal EEG theta activity linked with decreases in HPC-PFC theta coherence led us to hypothesize that there is a significant decrement in the amount of trajectory information processed and transferred between both HPC and PFC structures. This change would affect a communication channel between PFC and HPCs, through which relevant information would flow.

The template-misfolding of hyperphosphorylated tau can seed further protein aggregation along anatomically connected brain regions leading to a systematic and chronological progression of disease pathology. At 20 weeks post-PPF injection, tau pathology was significantly increased in hippocampal areas across both hemispheres, although the largest effect was observed in the injected hemisphere as previously described^37^. This is particularly relevant as the spread of tau aggregates may impair coherent activity ipsilateral and contralateral in the PFC-HPC circuit. Interestingly, the decrease in theta frequency coherent activity for inter-hemispherical CA1 was much slower than other regions as it started at 10 to 14 weeks post-PPF injection (Fig. 3a,b). Irrespective of connectivity, our functional data supports the notion that certain brain areas are more vulnerable to developing tau pathology than others, which may depend on intrinsic cell properties. Thus, it is likely that the shift in the power and functional connectivity strength towards the left hemisphere represent a driven compesantory factor in the K18 seed mice.

### MMN

Working memory, a fundamental construct of the temporary holding and manipulation of information is important for a range of executive cognitive tasks such as learning, comprehension and reasoning, and is often studied via mismatch negativity of event related brain potentials, which is a memory-based change-detection brain response to multi-stimulus pattern regularity^59^. The frontal cortex and the hippocampus are essential structures for working memory^60^, and neuronal activity in the PFC becomes synchronized with HPC neuronal activity during working memory tasks, and the strength of HPC-PFC synchrony as well as their coupling strength, is correlated with successful performance in cognitive tasks in animals^61^ and humans^62^. MMN is believed to provide a unique window to neuronal synchronization in processing infrequent and rare sound inputs^59,63^. Deficit in automatic auditory change detection that contributes to impairment in their ability to selectively attend and respond to deviant auditory stimuli has been described in MCI and AD patients^64,65^. Here, P301L tau seeded mice had disrupted P1/N1/P2 complex amplitude by reduced response to acoustic standard stimuli suggesting impairments of encoding or new learning, whereas the lowered MMN response to deviant stimuli may indicate impairments of recall and discrimination of deviant acoustic stimuli.

AD-related changes of oscillations may have some similarity to EEG changes during normal aging, as the amplitude of evoked gamma activity is decreased in aged healthy individuals^66^, and were correlated with lower clinical memory measures and behavioral performance in cognitive tasks^67^. A significant reduction or loss of 40 Hz gamma band synchronization in the thalamocortical areas has been described in AD patients^16,68^, in aging mice^69^ as well as in Aβ mouse model of AD^70^. In the present study, K18-P301L seed mice had consistent reduction in the gamma oscillations 40–80 Hz in response to both standard and deviant stimuli, whereas stronger oscillations coordinate successfully encoded working memory information on standard and deviant trials in buffer treated mice. Thus, the weaker coordinated evoked gamma oscillations may contribute to failure of P301L-K18 injected mice to encode and discriminate on standard and deviant trials, respectively.

### Pathology

The P301L AD mouse model is known to spontaneously develop tau pathology starting around 12 months of age, which is first apparent in the hippocampus and then progresses to the cerebral cortex. Consistent with our earlier study, in which K18 seeded P301L mice expressed a maximum tau pathology at week 4 and neuronal loss at week 8 after K18 seeding in the HPC^37^, the present work shows elevated AT8 immunoreactivity in HPC and entorhinal cortex suggesting maximal pathology load. Moreover, our experimental design allows for valid comparisons between the ammonium acetate buffer and K18 treated animals, as any possible effect of the buffer per se on putative neurotoxicity would have been present in both treatments^71^. Importantly, pathological tau is believed to spread transcellularly in a manner like prions in an anatomically defined pattern associated with progressive neuronal loss^72,73^. Synapse loss can be elicited either by the failure of neurons to maintain functional axons and dendrites, or by neuronal death^74^. In AD, with its slow progression, early synapse pruning precedes neuronal loss by several decades as shown in patients with mild cognitive impairment and early AD^75,76^. Inflammatory microglia activation contributes significantly to neuronal, structural and functional networks alterations^30,77^. GABAergic fast-spiking interneurons play a critical role in the synchronization of rhythmic oscillations and the organization and function of neuronal circuits, which are hypothesized to enhance information processing^78^. Interneuron dysfunction has been implicated in neurological and psychiatric disorders^39,79^. A dramatic early decrease in the number and complexity of septohippoampal GABAergic interneurons has been found in the APP mouse model^33^, and an amyloid beta injection in the hippocamapus greatly weakened the activity of rhythmic bursting septohippocampal GABAergic PV interneurons in the absence of detectable cells loss^80^. In the present study, the proportion of GABAergic cells was not different in different brain areas between controls and K18 injected mice. We therefore hypothesized that K18 injected in the hippocampus would perturb connectivity and functional PAC measures by reducing burst activity of GABAergic neurons through long lasting activation of inflammatory microglia that we found in the model^37^.

### Implications and conclusion

Dysfunction in neural networks has been commonly found in the brains of AD patients, and even observed before memory deficits became apparent in older, non-demented subjects with amyloid deposition^81^, suggesting this to be an early event in the pathogenesis of AD. Both increased and decreased neuronal excitability has been described in AD patients and in animal models of AD. On the one hand, hippocampal hyperactivity has been observed in patients with mild cognitive impairments^40^, the earliest symptomatic stage and even the asymptomatic preclinical stage^41^. On the other hand, neuronal activity declines in the later stages of AD, which has been correlated with a worsening of clinical symptoms in the elderly^82^. The present study provides direct evidence of tau seed-induced early dysfunctional network connectivity. The ability to directly seed and record from the HPC injection site was critical in this study as the contralateral was left non-seeded.

AD is a disconnection syndrome manifested by the disruption of white matter integrity, loss of synapses and functional connectivity across different cortical and subcortical regions. Synapses are an early site of dysfunction/pathology in AD^83^. Synaptic plasticity is strongly supported by the coupling of theta and gamma oscillations, pointing towards a potential function for the weaker theta-gamma coupling in the HPC and connectivity in the PFC-HPC circuit, of which early functional markers are sensitive to disease progression and can be used to monitor the severity of AD. Our findings in P301L mice treated with K18 showed early decreases of the EEG spectral power and no signs of epileptiform activity, which seems consistent with no direct role of tau in mediating dendritic hyperexcitability and epileptiform activity. The findings convincingly argue towards a causal relationship between the early disruption in functional networks and tau aggregation in the absence of Aβ pathology. Early indicators of AD are crucial for implementing interventions whilst the brain systems are still relatively normally functioning. The induction of intracellular tau aggregation by tau seed injection results in early altered network oscillations, connectivity and strong phase-amplitude theta–gamma oscillations uncoupling, which may be exploited as an electrophysiological translational marker to track early events of tau pathogenesis in taupathy and related disorders such as AD.

## Methods

### Animals and surgical procedure

All experiments were conducted in strict accordance with to the guidelines of the Association for Assessment and Accreditation of Laboratory Animal Care International (AAALAC), with the European Communities Council Directive of 24th November 1986 (86/609/EEC) and with protocols approved by the local Institutional Animal and Use Ethical Committee. Transgenic (Tg tauP301L) mice, expressing the longest human tau isoform with the P301L mutation (tau-4R/2 N-P301L) under control of the thy1 gene promoter^84^ were used for surgery at the age of 3 months. No gender differences were evident from earlier study^37^, hence only male mice were used in the present work. Animals received a microchip for identification purpose by an Animal Inventory and Weighing (AIW) system controlling animal health and well-being, and were housed in full-view plexiglas cages (25 × 33 cm, 18 cm high) that belong to IVC-racks (individually ventilated cages) located in a sound-attenuated chamber maintained under controlled environmental conditions (22 ± 2 °C ambient temperature, at 60% relative humidity, 12:12 light-dark cycle (lights on at 12 a.m. for polysomnographic, network connectivity and auditory mismatch negativity studies, light intensity: ~ 100 lux, food and water available ad libitum).

Truncated human tau fragments containing the four-microtubule binding repeat domain (K18; residues Q244-E372 of the longest human tau isoform) with a P301L mutation were produced in Escherichia coli (Tebu-bio, Le Perray-en-Yvelines, France). The fragments are flanked by a Myc tag at their C- or N-terminus. To obtain tau PFFs, tau K18-PL fragments (66 μM) were incubated at 37 °C for 5 days in the presence of 266 μM heparin (MP Biomedicals, Illkirch, France). The fibrillization mixture was centrifuged at 100,000 × *g* for 1 h and the resulting pellet was resuspended in 100 mM ammonium acetate buffer (pH 7.0) and sonicated before stereotaxic injection. Successful tau fibrillization was confirmed using Thioflavin T fluorescent assay (Sigma). Both buffer and (PFF) (25 µg/5 µl/0.5µlmin-1) were unilaterally (right hemisphere) injected into the hippocampus (AP − 2.5 mm from bregma, L + 2.0 mm, DV − 2.4 mm from the dura), respectively^37,73,85^.

The surgery was carried out according to the protocol described earlier^86^. Under isoflurane inhalation anesthesia, animals were stereotaxically equipped with 6 stainless steel fixing wires for the recording of EEG from frontal cortex (AP + 2 mm, L ± 1.4 mm from bregma), hippocampal CA1 (AP −2 mm, L ± 1.7 mm, DV − 1.8 mm from the dura) and CA3 (AP + 2.8 mm, L ± 3.2 mm, DV − 3.0 mm from the dura). All electrodes were referenced to the same ground electrode place midline above of the cerebellum. The incisor bar was −5 mm under the center of the ear bar, according to the stereotactic atlas of Paxinos and Franklin^87^. Electrodes were connected to a pin (Future Electronics: 0672-2-15-15-30-27-10-0) with a small insert (track pins; Dataflex: TRP-1558-0000) and were fit into a 10-hole connector then the whole assembly was fixed with dental cement to the cranium.

### Experimental design, recording and analysis procedures

#### Sleep-wake EEG spectral activity

Fast Fourier Transform analysis was used to compute the EEG power (µV^2^) in the EEG frequencies ranging from 1 to 50 Hz in consecutive artefact-free 4 s epochs of wakefulness, NREM and REM sleep over 24 hours (See supplementary method). Changes in EEG spectral power profiles were expressed as the mean relative spectral profiles in buffer and K18-treated mice during each 24 hrs recording session. Subsequent log-transform analysis assessed longitudinal changes in specific frequency bins across different vigilance states.

#### EEG network oscillations and connectivity study

EEG recordings were performed under vigilance controlled waking condition during the dark phase as described earlier^85^. General motor activity was measured in the home cage by two passive infrared (PIR) detectors placed above each recording cage, and the envelope activity was used to analyze motion levels. Only continuous waking artefact-free 4-second epochs with low-voltage fast EEG activity and high to moderate body activities were used in the analysis. A notch finite impulse response filter at 50 Hz was applied to avoid voltage related to power line interferences. EEGs were recorded for 3 hrs at 2 kHz sample rate with a Biosemi ActiveTwo system (Biosemi, Amsterdam, Netherlands), digitized with 24-bit resolution and band-pass filtered between 1 and 100 Hz. Analysis was performed using a method described in detail earlier^85^. In brief, spectral density estimates were calculated in blocks using Fast Fourier Transform with Hanning window function, and power was expressed as percentage of total power over 1–100 Hz. The average spectral power density in each frequency bin of each brain location was averaged across animals to obtain the full power spectrum (spectrogram with 1 Hz resolution in frequency domain and 4 s resolution in time domain) constructed from overlapping windows of 50% for 4 sec data and plotted for baseline periods and post-drug periods windowing over 1–100 Hz. Buffer and K18-induced changes in EEG spectral power was calculated in 3 hour blocks as the ratio of mean spectral power obtained in each recording session versus the mean spectral power obtained during the first recording session. The procedure quantifies longitudinal K18 versus buffer-induced changes in different frequency bands as a percentage of the original power.

#### Phase-Amplitude Cross-Frequency Coupling

The nonlinear phase-amplitude coupling (PAC) was longitudinally calculated using the algorithm described previously^52^ and the derived modulation index (MI) estimates indicate how the buffer or the PFF K18 affects the strength of interactions between phases of low-frequency oscillations and amplitudes of higher-frequency oscillations. PAC values were assessed for each EEG electrode in 45 min length intervals starting at 15 min from the onset of each recording session. MI was estimated between low frequencies (“phase”) f_L_ taken from interval 2–12 Hz with a step of 2 Hz and frequencies (“amplitude”) f_H_ taken from interval 10–200 Hz with a step of 5 Hz. MI index is reported as absolute value of mean (over time) of complex-valued signal z(t) = A_H_(t)∙exp(i∙φ_L_(t)), where t is time, A_H_(t) is analytic amplitude obtained via Hilbert transform of narrow band pass signal x_H_(t) centered around frequency f_H_, and φ_L_(t) is analytic phase obtained via Hilbert transform of narrow band pass signal _xL_(t) centred around frequency f_L_.

#### Network connectivity through Coherence

Longitudinal effects of K18 on the integrity of cortical neural pathways and the functional coupling between different cortical structures at various frequency oscillations were estimated according to the procedure described earlier^85^. Coherence is reported as normalized values between 0 and 1: a low value indicates no similarity between the two signals, whereas values close to 1 indicate a high similarity between two-time series signals up to near constant phase shift. The coherence function, which gives information on the stability of the similarity at each frequency bin between the time series in different electrodes, was quantified at different time points by dividing the numerical square of the absolute value of the cross-spectrum by the product of the autospectra: Coh(f) = |S_AB_(f)|^2^/(S_AA_(f)S_BB_(f)); where S_AB_ is the cross-spectral density between the signals A and B; S_AA_ is the autospectral density of the signal A; S_BB_ is the autospectral density of the signal B. Coherence index is sensitive to a change in power and a change in phase relationships. Subsequently, coherence spectra were calculated for every Buffer and K18 conditions and pair of derivations at every 4 sec and aggregated over 15–60 min in each frequency bin for each animal, time point, and treatment group (buffer vs K18).

#### Auditory evoked potentials (AEPs): Mismatch negativity (MMN) paradigm

The passive oddball MMN response was assessed by recording a stable baseline EEG during 20 min without acoustic stimuli followed by 2 runs of 20 min auditory evoked potential (AEP) responses, of which auditory stimuli were delivered through a speaker mounted above the floor of the experimental box. A series of 240 frequent standard 2 KHz tones (probability 80%) and infrequent 60 oddball deviant 4 KHz tones (probability 20%), with intensity 87 dB SPL and 20-ms duration with interstimulus interval of 4 s. Intervals of EEG recordings spanning from 50 ms before to 450 ms after stimulus onset were extracted, baseline corrected and averaged ERP waveforms were computed for each animal and stimulus condition, whilst the average across animals resulted in grand average ERPs. The peak amplitudes and latencies of the P1, N1, P2 and N2 components of the ERPs were calculated for each subject and averaged for each condition. Additionally, spectrograms of event-related oscillations were estimated using Morlet wavelet analysis for each stimulus condition and treatment group.

#### Immunohistochemistry and validation of K18 injection sites and EEG electrodes placement

Mice were sacrificed at the end of the *in vivo* study by decapitation, brains were rapidly removed from the skull and immersion fixed in formalin-based fixative to minimize post-mortem dephosphorylation. Quantification of hyperphosphorylated tau was performed according to the protocol described earlier^37^. Briefly, brains were extirpated postmortem, immersed in formalin-based fixative overnight, embedded in paraffin and sliced at 5 µm. Deparaffinization and rehydration of the sections was followed by heat induced antigen retrieval in citrate buffer (pH 6.0) and quenching of endogenous peroxidase activity in 3% hydrogen peroxide solution. Samples were incubated for one hour with primary antibodies AT8, which recognizes tau phosphorylated at Ser202/Thr205 (in-house made, 0,2 µg/ml) or anti-Parvalbumin (Swant, Marly, Switserland, PV235, 1/1000), diluted in antibody diluent with background reducing components (DAKO, Glostrup, Denmark). After several washing steps, HRP tagged anti-mouse secondary antibody (Envision, DAKO) was applied for 30 minutes, followed by chromogenic labelling with 3,3-diaminobenzidine (DAB)(DAKO). Sections were counterstained with hematoxylin, dehydrated and mounted (Vectamount, Vector Labs, Burlingame, CA, USA). Stained sections were scanned with a NanoZoomer slide scanner 2.0 rs (Hamamatsu Photonics, Shizuoka, Japan). Regions-of-interest (ROIs) were manually delineated in accordance with the Franklin and Paxinos atlas 86 and for each ROI the percentage of DAB-positive area/total area was calculated in Matlab/Phaedra.

PV+ labelling was quantified as % DAB positive area/total region-of-interest (cortex or entorhinal cortex). Regions were manually delineated in the analysis program (Matlab/Phaedra), according to the Franklin and Paxinos atlas. Threshold for DAB+ labelling was set at 0.5 ((R − G)/B = 0.5, with R, G and B the red, green and blue intensity between 0 and 1), including only parvalbumin positive cells for the analysis.

Histological verification of electrodes and the injection sites were carried out in brain sections (20 µm) that were stained. Data from animals that did not meet strict EEG electrode placement criteria and the injection site localization were excluded from the study.

#### Statistical Analysis

Comparisons of response variables between treatment groups were estimated from linear mixed models. The hierarchical structure of the data (multiple observations within animals from different time points and/or brain regions) was taken into account by including animal as a random factor in the models. Residual errors were assumed to be normally distributed. Adjustments for multiple testing were done using Hochberg’s and Dunnett’s (EEG spectra) or Bonferroni’s (immunohistology) procedures. Confidence interval and Student t-test for independent samples were used to estimate differences in coherence values between buffer and K18 groups. For PAC MI values, normalization and statistical assessment (α = 0.05) were done through construction of 50 surrogates and using Bonferroni correction^52^. Only statistically significant results were reported on comodulogram maps. The critical level for significance was set to 0.05. All significance tests were two-tailed.

### Data Availability

All data generated or analysed during this study are included in this published article (and its Supplementary Information files).

## Electronic supplementary material

Supplementary Information
