# Supplementary material for: Emergence of early alterations in network oscillations and functional connectivity in a tau seeding mouse model of Alzheimer’s disease pathology
Source: Sci Rep. 2017 Oct 27;7:14189. doi: 10.1038/s41598-017-13839-6 (PMC5660172; doi:10.1038/s41598-017-13839-6)
Supplement: Supplementary file 1 — Supplementary Information [file 41598_2017_13839_MOESM1_ESM.pdf]

Emergence of early alterations in network oscillations and functional connectivity in a tau seeding mouse model of Alzheimer's disease pathology

Ahnaou A, Moechars D, Raeymaekers L, Biermans R, Manyakov NV, Bottelbergs A, Wintmolders C, Van Kolen K, Van De Casteele T, Kemp JA and Drinkenburg WH

Supplementary

Materials and methods

Surgery procedure for sleep-wake cycle

EEG, BT/LMA recordings and vigilance state analysis. The surgical procedure was performed according to the procedure described earlier.[1] Under isoflurane anesthesia, mice were surgically implanted with a transmitter (TA10ETA-F20, Data Science International, USA) placed in the peritoneal cavity to allow the sensing of body temperature (BT) and locomotor activity (LMA) longitudinally. The leads of the probe were tunneled from the peritoneal cavity and led subcutaneously to the head in order to fix the end tip of the lead to stainless screws for longitudinal epidural electroencephalographic (EEG) monitoring. The incision in the abdominal wall was sutured with uninterrupted stitches and epidural screws were fixed into the skull, covered with dental cement and the skin above the head closed with nylon sutures.

After surgery, the animals received, subcutaneously, 0.3 ml analgesic (Carprofen, Rimadyl, 50mg/ml, Pfizer Ltd, UK diluted 1:10) and local analgesic on the wounds (Lidocaine spray Xylocaine, 1% solution, Astra Pharmaceuticals Ltd, UK). Subsequently, animals were individually placed in their home cage and kept warm in a heating box set at 26 °C ± 2 °C to avoid hypothermia with the temperature progressively decreasing over days until it reached room temperature. The animals were allowed to recover from surgery for at least 2 weeks.

Recording and analysis

Recordings of spontaneous sleep-wake cycles over 24 hours were performed as described earlier[1]. Every two weeks over a 5-month period animals were placed, whilst remaining in their home cages, on top of the appropriate receivers and telemetry signals were processed for analogue output

by a Data Sciences International analogue converter (Dataquest ART 2.3 Gold version). BT sampled for 10-sec was averaged at 5 min intervals and movements in the cage was accounted in 5-minute bins. The signals were digitized at a sampling rate of 200 Hz, imported offline into Neuroscore software (Neuroscore, DSI) and digitally band pass filtered between 0.5 and 50 Hz while analyzing the vigilance states. The vigilance states were scored in 4 s epochs as being either wakefulness, non-rapid eye movement (NREM) sleep or rapid eye movement (REM) sleep, based on EEG characteristics and locomotor activity (LMA).

Results

Vigilance states

Hourly profile of vigilance states, BT and LMA under baseline conditions are presented in Figure 1A (only data of 2, 4 and 8 weeks post-HPC infusion of K18 were displayed for the spontaneous sleep-wake architecture). The sleep-wake cycle was generally preserved with a higher amount of waking dominantly expressed during the dark phase, whereas NREM sleep and REM sleep were largely expressed during the light phase of the circadian time. Patterns of waking, NREM sleep, REM sleep, core body temperature, locomotor activity were qualitatively similar over 24 weeks recordings sessions between K18 and buffer-treated mice, while tau pathology reached its maximal level at 4 weeks post-injection and neuronal loss at 8 weeks post-K18 injection.[2] However, at week 16, K18-injected mice showed increases in the shift between waking and NREM sleep states during both light and dark periods of the circadian time, suggesting profound sleep instability (Figure 1b).

Discussion

Sleep-wake architecture

Sleep serves vital functions such as homeostatic restoration and synaptic plasticity, removal of waste products in the brain, and has a critical role in cognitive processing including memory triage and consolidation.[3-6] Consistent with the bidirectional association that exists between AD and the quality of sleep, key brain structures involved in the regulation of sleep and circadian rhythms are affected early in the pathogenesis. Sleep disturbances occur early in the course of AD and are found to precede the onset of cognitive symptoms in patients with AD, while sleep quality and/or circadian function declines further in parallel with progression of both cognitive dysfunction and AD pathology[7-9], and therefore NREM sleep has been suggested as a powerful noninvasive mechanistic pathway biomarker for an early diagnostic of the disease condition.[7] EEG abnormalities during sleep include fewer sleep spindles and reduced amounts of NREM sleep.

In mice models of AD, in which Aβ deposition develops in the brain, increased wakefulness associated with slowed EEG and decreased sleep duration starts around the time that amyloid plaques begin to accumulate in the hippocampus and cortex (6 months of age). Based on those observations, we expected that PPF aggregate would also disrupt sleep. P301L seed mice develop amyloid plaques starting around 6 months of age which is first apparent in the cortex and progresses to the hippocampus with age[10], while P301L mice develop maximal tau pathology at 3 weeks and neuronal loss at 8 weeks after the injection of the PPF in the hippocampus.[2] In the later model, quantification of sleeping and waking states did not reveal changes in the amount of sleeping and waking, nor in BT and LMA rhythms, however the increased transitions between waking and NREM state to indicate difficulties in the maintenance of sleep continuity and stability with possible consequences for accelerated tau pathophysiology.

Figure legend

Supplementary Figure 1: (A) Hourly changes in wakefulness, NREM sleep and REM sleep expressed (min), body temperature (°C) and locomotor activity (counts/min) over time for weeks 2, 4 and 8 post-administration in buffer (black, n=11, 4 animals were excluded due to mislocation of the injection and electrode sites or signal artefacts) and K18 condition (green, n=15). Black bar on the abscissa above the x-axes indicates the dark phase of the circadian time. (**B)** Number of transitions between vigilance states. Data are mean value ± SEM for buffer (black) and K18 (green) conditions. Mixed model ANOVA and * $p<0.05$ Student t test.

References

1. Ahnaou, A. et al. Modulation of group II metabotropic glutamate receptor (mGlu2) elicits common changes in rat and mice sleep-wake architecture. *Eur J Pharmacol* **603**: 62–72 (2009).

2. Peeraer, E. et al. Intracerebral injection of preformed synthetic tau fibrils initiates widespread tauopathy. *Neurobiol Dis* **73**, 83–95 (2015).

3. Bakker, E.N. et al. Lymphatic Clearance of the Brain: Perivascular, Paravascular and Significance for Neurodegenerative Diseases. *Cell Mol Neurobiol* **36**, 181–94 (2016).

4. Stickgold, R. & Walker, M.P. Sleep-dependent memory triage: evolving generalization through selective processing. *Nat Neurosci* **16**, 139–45 (2013).

5. Walker, M.P. Cognitive consequences of sleep and sleep loss. *Sleep Med* **9 Suppl 1**, S29–34 (2008).

6. Xie, L. et al. Sleep drives metabolite clearance from the adult brain. *Science* **342**, 373–7 (2013).

7. Mander, B.A., Winer, J.R., Jagust, W.J. & Walker, M.P. Sleep: A Novel Mechanistic Pathway, Biomarker, and Treatment Target in the Pathology of Alzheimer's Disease? *Trends Neurosci* **39**, 552–66 (2016).

8. Porter, V.R., Buxton, W.G. & Avidan, A.Y. Sleep, Cognition and Dementia. *Curr Psychiatry Rep* **17,** 97 (2015).

9. Swaab, D.F., Fliers, E. & Partiman, T.S. The suprachiasmatic nucleus of the human brain in relation to sex, age and senile dementia. *Brain Res* **342**, 37–44 (1985).

10. Oddo, S., Caccamo, A., Kitazawa, M., Tseng, B.P. & LaFerla, F.M. Amyloid deposition precedes tangle formation in a triple transgenic model of Alzheimer's disease. *Neurobiol Aging* **24**, 1063–70 (2003).

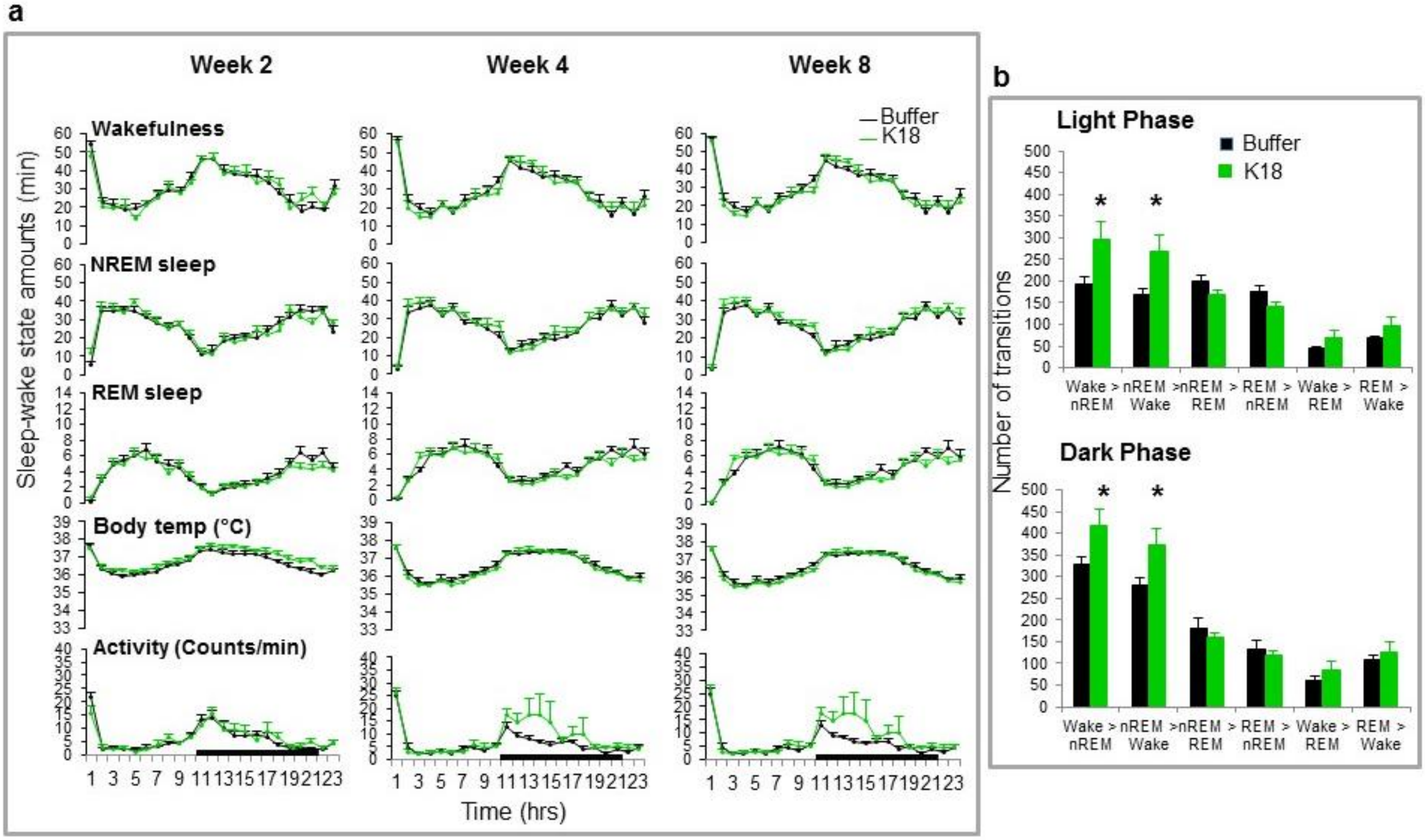
a
Week 2
Week 4
Week 8
Buffer
K18
Wakefulness
NREM sleep
REM sleep
Body temp (°C)
Activity (Counts/min)
Sleep-wake state amounts (min)
Time (hrs)
b
Light Phase
Dark Phase
Buffer
K18
Number of transitions
Wake > nREM
nREM > Wake
>nREM > REM
REM > nREM
Wake > REM
REM > Wake
